# Sphingolipidomics: An Important Mechanistic Tool for Studying Fungal Pathogens

**DOI:** 10.3389/fmicb.2016.00501

**Published:** 2016-04-14

**Authors:** Ashutosh Singh, Maurizio Del Poeta

**Affiliations:** ^1^Department of Molecular Genetics and Microbiology, Stony Brook University, Stony BrookNY, USA; ^2^Veterans Administration Medical Center, NorthportNY, USA

**Keywords:** sphingolipids, high performance liquid chromatography, electrospray ionization tandem mass spectrometry, fungi, fungal infections, ceramide

## Abstract

Sphingolipids form of a unique and complex group of bioactive lipids in fungi. Structurally, sphingolipids of fungi are quite diverse with unique differences in the sphingoid backbone, amide linked fatty acyl chain and the polar head group. Two of the most studied and conserved sphingolipid classes in fungi are the glucosyl- or galactosyl-ceramides and the phosphorylinositol containing phytoceramides. Comprehensive structural characterization and quantification of these lipids is largely based on advanced analytical mass spectrometry based lipidomic methods. While separation of complex lipid mixtures is achieved through high performance liquid chromatography, the soft – electrospray ionization tandem mass spectrometry allows a high sensitivity and selectivity of detection. Herein, we present an overview of lipid extraction, chromatographic separation and mass spectrometry employed in qualitative and quantitative sphingolipidomics in fungi.

## Introduction

As a group, sphingolipids are essential components of all eukaryotic cell membranes (Dickson and Lester, 2002). In fungi, sphingolipids play an important role in a variety of biological processes like cell division (Epstein et al., 2012), heat stress response (Jenkins et al., 1997), acid/alkaline tolerance (Luberto et al., 2001; Rittershaus et al., 2006), hyphae formation (Oura and Kajiwara, 2010), domain formation (Marquês et al., 2015) spore germination (Levery et al., 2002), endocytosis (Zanolari et al., 2000), signal transduction (Obeid et al., 2002), apoptosis (Cheng et al., 2003), pathogenesis and virulence (Luberto et al., 2001; Rittershaus et al., 2006). Endowed with unique chemical structure and synthesized by fungal specific enzymes, these sphingolipids are ideal drug targets (Mor et al., 2015). In this context, it is important to characterize sphingolipids in greater detail.

**FIGURE 1:**
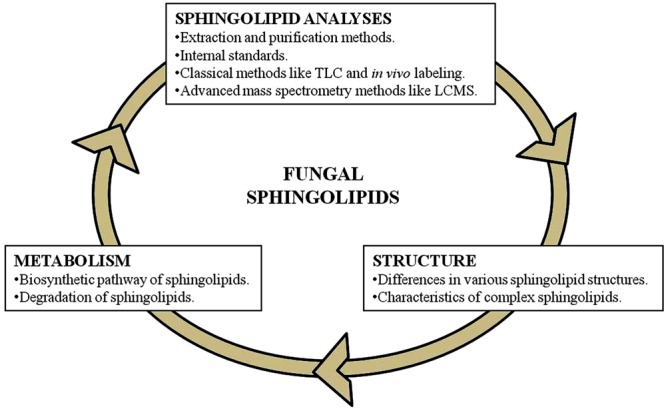
**Key components of sphingolipid characterization in fungi**.

Characterization of various fungal sphingolipids requires the understanding of three key components (**Figure 1**). These are: the pathways of biosynthesis and degradation; structures of various sphingolipids being synthesized; understanding the ways for efficient extraction of sphingolipids from the cell and the precise methods for their analysis. A tremendous amount of literature in the field of fungal lipid metabolism allows us to categorically understand these components. In the sections below, we have described a literature based review of these key components of sphingolipid characterization, with an emphasis on mass spectrometry based structural and functional characterization of sphingolipids from pathogenic fungi.

**FIGURE 2:**
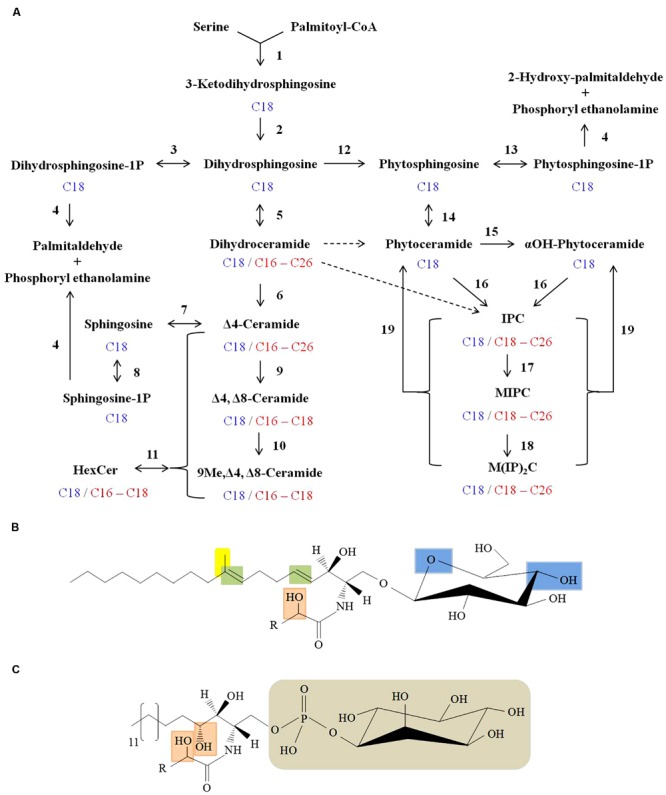
**Sphingolipid biosynthesis in fungi. (A)** Scheme of fungal sphingolipid biosynthetic pathway. Enzymatic steps with reverse reaction shown in brackets are as follows: (1) Serine *C*-palmitoyl transferase; (2) 3-ketosphinganine reductase; (3) Dihydrosphingosine kinase [Dihydrosphingosine-1P-phosphatase]; (4) Long-chain base (LCB) phosphate lyase; (5) Dihydroceramide synthase; (6) Dihydroceramide C4 desaturase; (7) Ceramidase [Ceramide synthase]; (8) Sphingosine kinase [Sphingosine-1P-phosphatase]; (9) Ceramide C8 desaturase; (10) Sphingolipid C9 methyltransferase (Smt1); (11) Hexosyl ceramide synthase [Hexosyl ceramidase]; (12) Dihydrosphingosine C4-hydroxylase (13) Phytosphingosine kinase [Phytosphingosine -1P-phosphatase]; (14) Ceramide synthase [Ceramidase]; (15) Sphingolipid α-hydroxylase; (16) IPC synthase; (17) IPC mannosyl transferase; (18) Inositolphosphotransferase; (19) Inositol phosphosphingolipid phospholipase C. Major sphingoid backbone in fungi is composed of 18 C-atoms (shown in blue) and the fatty acyls attached to various sphingolipids comprise of 16–26 C-atoms (shown in red). Putative conversion steps of dihydroceramide to phytoceramide and to IPC are represented by dashed arrows. **(B)** Structure of fungal glucosylceramide. Long chain sphingoid backbone amide linked to a fatty acyl and linked by β-glycosidic bond to a polar head group (glucose) at C1 position. Unique features of fungal glucosylceramide are: Δ4 and Δ8 double bonds in the sphingoid backbone (shown in green), 9-methylation in the sphingoid backbone (shown in yellow), α-hydroxyl fatty acyl (shown in orange) and hydroxyl groups in the hexose moiety (shown in blue). **(C)** Structure of fungal IPC. The distinguished features of IPC’s in fungi are: C3-hydroxylation of sphingoid backbone and α-hydroxyl fatty acyl (both shown in orange), phosphate linked inositol group (shown in grey). ‘*R*’ represents 16–24 carbons in the fatty acyl chain.

In general, the structure of sphingolipids comprise a LCB backbone amide- linked to a fatty acid at C2 position and ester linked to a polar head group at C1 position (Del Poeta et al., 2014). There is a large diversity in sphingoid bases of mammals (Kendall et al., 2015), plants (Luttgeharm et al., 2015), and fungi (Del Poeta et al., 2014). In fungal cells, *de novo* sphingolipid biosynthesis begins with condensation of *L*-serine and palmitoyl-CoA to form 3-ketodihydrosphingosine which is then reduced to dihydrosphingosine (d18:0 backbone; sphinganine; **Figure 2A**; Buede et al., 1991; Nagiec et al., 1994). This step leads to the formation of an 18 C containing LCB. Dihydrosphingosine is then amide linked to a fatty acid (usually α-hydroxylated, 18 or 16 C containing) by ceramide synthases to form dihydroceramide (Lahiri and Futerman, 2005). Further, a Δ4-desaturation of the sphingoid backbone of dihydroceramide forms ceramide (d18:1 backbone; 4-sphingenine; Ternes et al., 2011; Rodriguez-Cuenca et al., 2015). Next, a Δ8-desaturation of the sphingoid backbone of Δ4-ceramide leads to formation of Δ4, Δ8-ceramide (d18:2 backbone; 4,8-sphingadienine; Sperling et al., 2000; **Figure 2A**). A sphingolipid C9-methyl transferase catalyzes the addition of a methyl group at C9 position of the 4,8-sphingadienine base of Δ4, Δ8-ceramide to form 9-methyl-Δ4, Δ8-ceramide (d19:2 backbone; 9-methyl-4,8-sphingadienine; Ternes et al., 2006; Singh et al., 2012; **Figure 2A**). Finally, a Gcs1 catalyzes the transfer of glucose moiety from the UDP-glucose onto the C1 hydroxyl group of the ceramide forming glucosylceramide (Rittershaus et al., 2006). Although all the enzymatic steps are not well characterized, some have been studied in fungi like *Cryptococcus neoformans* (Rittershaus et al., 2006; Singh et al., 2012), *Aspergillus nidulans* (Li et al., 2006), *A. fumigatus* (Levery et al., 2002; Kotz et al., 2010), *Fusarium graminearum* (Rittenour et al., 2011) and *Candida albicans* (Cheon et al., 2012). Notably, glucosylceramide biosynthesis is absent in *Saccharomyces cerevisiae* and *C. glabrata* (Leipelt et al., 2001).

In plants, 8-sphingenine (d18:1) and 4,8-sphingenine (d18:2) represent the major sphingoid bases, however, presence of the *cis-* and *trans-* isomeric forms result in nine different C18 sphingoid bases (Ohnishi et al., 1983). This results in a complex mixture of glucosylceramide pool in plants (Imai et al., 2012). In mammals, the major sphingoid base is 4-sphingenine (d18:1) which is linked to 16 C fatty acid in the glucosylceramide structure (Leipelt et al., 2001). In contrast, the fungal glucosylceramide structure is rather unique. The sphingoid backbone is composed of 9-methyl-4,8-sphingadienine which is amide linked to α-hydroxylated C18:0 fatty acid (**Figure 2B**; Del Poeta et al., 2014). Certain fungal species contain galactose instead of glucose in this cerebroside structure (Warnecke and Heinz, 2003). The α-hydroxylated C16:0 and α-hydroxylated C18:1 are the two other major fatty acyls reported in the fungal cerebroside structure (Barreto-Bergter et al., 2004). The structures of different cerebrosides produced by fungi have been extensively reviewed by Barreto-Bergter et al. (2011).

Fungi also produce phytoceramide (Heung et al., 2006). Dihydrosphingosine C4-hydroxylase catalyzes the addition of a hydroxyl group at C4 position of dihydrosphingosine backbone to produce phytosphingosine (t18:0 backbone; 4-hydroxysphinganine; Li et al., 2007; **Figure 2A**). Ceramide synthases then transfer a non-hydroxylated fatty acid (18, 24 or 26 C containing) to amide group at C2 position to form phytoceramide (Cheon et al., 2012). The fatty acyl moiety of the phytoceramides is α-hydroxylated by a sphingolipid α-hydroxylase to form αOH-phytoceramides (Haak et al., 1997). Both phytoceramides and αOH-phytoceramides are further converted to complex phosphosphingolipids such as IPC (transfer of phosphorylinositol group to phytoceramide), MIPC, (transfer of mannosyl group to IPC) and M(IP)_2_C, (transfer of a second phosphorylinositol group to MIPC; Guan and Wenk, 2008). Although IPC derivatives are not reported in mammals, certain plants and kinetoplastid protozoa do produce IPC derivatives (Heung et al., 2006).

The t18:0 phytosphingosine backbone, C2 hygroxylated fatty acyls and phosphorylinositol containing polar head group are the unique feature of IPC derivatives (**Figure 2C**). IPC structures and pathway of synthesis have been characterized in several fungi like *S. cereviseae* (Ejsing et al., 2009; Voynova et al., 2014), *C. neoformans* (Luberto et al., 2001), *A. fumigatus* (Kuroda et al., 1999; Heidler and Radding, 2000), *A. nidulans* (Bennion et al., 2003), *Histoplasma capsulatum* (Guimarães et al., 2014), and *C. albicans* (Singh et al., 2010; Cheon et al., 2012).

The re-cycling of fungal sphingolipids involves the production of sphingosine, sphingosine-1-phosphate, dihydrosphingosine-1-phosphate, and phytosphingosine-1-phosphate (**Figure 2A**). The ultimate degradation steps of sphingolipids are constituted by the catabolism of dihydrosphingosine-1-phosphate and sphingosine-1-phosphate into palmitaldehyde and phosphoryl ethanolamine (Kim et al., 2000; Serra and Saba, 2010) and the phytosphingosine-1-phosphate into 2-hydroxy-palmitaldehyde and phosphoryl ethanolamine (Kondo et al., 2014), by the long-chain basse-1-phosphate lyases (**Figure 2A**).

The ability of fungal cells to produce sphingolipids with different sphingoid backbone structures adds to the complexity of the lipid mixtures (Guimarães et al., 2014). In addition to the backbone structure, the degree of unsaturation, fatty acyl chain lengths, methylation and hydroxylation modifications, all make it quite difficult to analyze these lipids using classical techniques like *in vivo* labeling (Chigorno et al., 2006), TLC (Urban et al., 1980) and GC (Cacas et al., 2012). Therefore, advanced analytical methods for the analyses of these lipids are employed to characterize their structures.

In last two decades, several mass spectrometry techniques have been used to identify and characterize the total sphingolipid pool or the “sphingolipidome.” A wide variety of mass spectrometry based methods are available in literature that allows accurate analyses of these complex sphingolipid mixtures (Merrill et al., 2005, 2009; Haynes et al., 2009; Wenk, 2010; Köfeler et al., 2012). Below we describe methods of extraction, chromatographic separation and the mass spectrometry based strategies to analyze sphingolipids.

## Lipid Extraction

**FIGURE 3:**
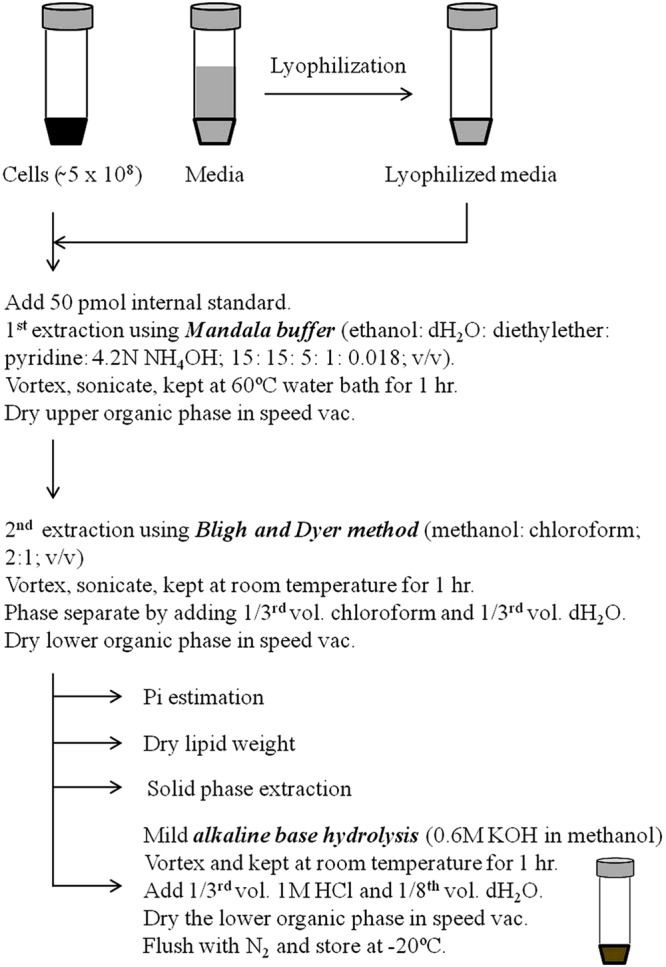
**Procedure of sphingolipid extraction.** Prior to extraction, fungal cell extracts or lyophilized media are spiked with suitable internal standards. First extraction is performed using Mandala extraction buffer (ethanol: dH_2_O: diethyl ether: pyridine: 4.2N NH_4_OH; 15: 15: 5: 1: 0.018; v/v). Subsequently, a second Bligh and Dyer extraction is performed using methanol and chloroform. Samples at this stage can be used to determine the Pi content, lipid dry weight or to purify specific sphingolipid components using solid phase extraction. Finally, samples are base hydrolyzed using mild alkaline base hydrolysis (0.6 M KOH in methanol) to remove the glycerol backbone containing lipid contaminants. For certain fungal masses it is required to first crush the sample using glass beads in the lipid extraction buffer itself or to make the sample into powder using pestle and mortar in liquid N_2_ prior to extraction.

Extraction of lipids is the most crucial step for lipid analysis by both classical and high throughput techniques. Currently several modified adaptations of Folch method (Folch et al., 1957) and Bligh and Dyer method (Bligh and Dyer, 1959) are being employed to extract lipids from fungal cells (Prasad and Ghannoum, 1996; Schneiter and Daum, 2006; Ejsing et al., 2009; Haynes et al., 2009). In our laboratory and others, we use the method described by Mandala et al. (1995) for lipid extraction. This method has shown a good extraction efficiency and reproducibility for lipid analyses. The scheme of lipid extraction from fungal cells is shown in **Figure 3**. Fungal cells are extracted in ethanol: dH_2_O:diethylether:pyridine:NH4OH (15:15:5:1:0.018; v/v) at 60°C for 1 h as described previously (Hanson and Lester, 1980). Lipid extract is then subjected to a solution of methanol:chloroform (2:1; v/v) followed by addition of 1/3rd volume chloroform and 1/3rd volume dH_2_O and the lower organic phase reserved (Bligh and Dyer, 1959). Organic phase is dried in SpeedVac, flushed with N_2_ and stored in -20°C. At this stage, these lipid extracts can be used for the estimation of inorganic phosphate (Pi) content, dry lipid weight or for the isolation and purification specific lipid classes (Merrill et al., 2009; Rana et al., 2015). Both Pi content and dry lipid weight have been used to normalize the lipid amounts. Several groups have employed SPE techniques to further purify specific sphingolipid groups (Bodennec et al., 2000; Barreto-Bergter et al., 2004). Extensive lipid purification by SPE is cumbersome and time consuming, and is usually not required for routine lipid analysis; however, is preferred for a thorough structural characterization of complex glycosphingolipids (Barreto-Bergter et al., 2004, 2011).

Glycerolipids are the major lipid contaminants that are co-extracted with sphingolipids (Bligh and Dyer, 1959). A mild alkaline methanolysis (Clarke and Dawson, 1981), usually for 60 min at room temperature is sufficient to hydrolyze the ester linkages of fatty acyls of glycerolipids. This allows a clean extraction of alkaline-stable components, which are highly enriched in sphingolipids.

For accurate quantification of lipids by mass spectrometry, internal standards must be added prior to lipid extraction (Rana et al., 2015). Although, absolute quantification of each lipid species by mass spectrometry requires the use of isotope-labeled standard for that species (Ecker and Liebisch, 2014); however, presently this is not possible due to high synthesis costs and the large number of lipids being analyzed. Addition of one internal standard per lipid class being analyzed is widely accepted (LIPID MAPS Consortium). This is primarily because the ionization of lipid species is largely dependent upon the specific head group rather than the attached fatty acyls (Köfeler et al., 2012). The C17-sphingoid backbone lipids (Avanti Polar Lipids Inc., Alabaster, AL, USA; Matreya Inc., Pleasant Gap, PA, USA) are routinely used as internal standards as these closely resemble C18 lipids in physicochemical properties and ionization efficiencies (Rana et al., 2015). Quantification of sphingolipid species with different chain lengths can be achieved using the calibration curves of closest chain length standards (Rana et al., 2015).

## Methods of Lipid Analysis

### *In Vivo* Labeling, Thin-layer Chromatography and Gas Chromatography Mass Spectrometry

For several decades, *in vivo* labeling has been used to characterize the sphingolipid metabolic pathways in fungi (Chigorno et al., 2006). The technique involves the uptake of a radiolabeled precursor by cells. The radiolabeled precursor then gets incorporated into the complex lipid structures. Heavy labeled [^14^C]-palmitate and [^14^C]-serine are the most commonly used labeled precursors to follow sphingolipid synthesis (Chigorno et al., 2006). However, these labels get incorporated into other untargeted lipids and give high background signals. [^3^H]-dihydrosphingosine provides a more sphingolipid specific labeling (Karashima et al., 2013). [^3^H]-inositol has been used to focus on IPC derivatives (Haak et al., 1997) and [^3^H]- or [^14^C]- glucose or galactose have been used to focus on glycosphingolipids (Sasaki, 1981). Unfortunately, the very long half-life of these radiolabeled precursors presents a serious risk to health if exposed and is a challenge during disposal (Ecker and Liebisch, 2014).

**FIGURE 4:**
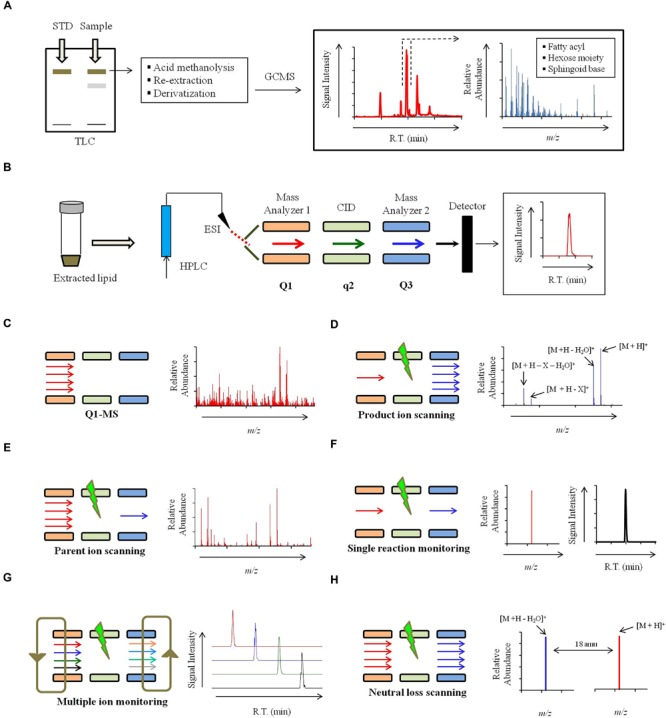
**Strategy for mass spectrometry based identification of sphinglipids. (A)** Alternate strategies to confirm the sphingolipid structure. Qualitative analysis of sphingolipids can be performed using commercially available standards by High performance thin-layer chromatography (HP-TLC) or TLC. Sphingolipid components like sphingoid backbone, sugar and fatty can be analyzed using Gas chromatography mass spectrometry (GCMS). For sphingoid base and sugar analysis, samples are prepared using following steps: acid methanolysis (1 N HCl in methanol), *N*-acetylation (pyridine and acetic anhydride) and per-*O*-trimethylsilylation [TriSil reagent; 1-(Trimethylsilyl)imidazole – Pyridine mixture]. Sphingoid bases and sugars are analyzed as *N*-acetyl-per-*O*-trimethylsilyl derivatives and monosaccharide methyl glycosides, respectively. For fatty acyl analysis, samples are prepared using acid methanolysis, re-extraction in *n*-hexane and per-*O*-trimethylsilylated (TriSil) to form fatty acid methyl esters (FAMEs). **(B)** A scheme of HPLC-ESI-MS/MS. Lipids are resolved by HPLC and thereafter analyzed by tandem mass spectrometry (MS/MS). ‘Q1’ and ‘Q3’ quadruples are mass analyzers and the quadrupole ‘*q*’ operates in radio frequency leading to collision-induced dissociation (CID). Electrospray ionization (ESI) or Atmospheric pressure chemical ionization (APCI, not shown) is used as ionization methods. **(C)** Scheme for Q1-MS or full scan. The molecular ions or *m/z* ratio are recorded in Q1. **(D)** Scheme for product ion scanning. Only a select ion with specific *m/z* is recorded in Q1 and undergoes CID. Resultant ions are recorded in Q3. ‘*M*’ represents the molecular ion; ‘*X*’ represents a fragment loss. **(E)** Scheme for parent ion scanning. A full scan of ions recorded in Q1 and following CID, only a select fragment ion is recorded in Q3. **(F)** Scheme for single-reaction monitoring (SRM). A preselected ion is recorded in Q1, a select parent ion undergoes CID and a select fragment ion is recorded in Q3. **(G)** Scheme for multiple-reaction monitoring (MRM). Multiple SRM’s can be set up simultaneously depending upon the scanning efficiency of the instrument. **(H)** Scheme for neutral loss (NL) scanning. Ions are recorded in both Q1 and Q3 with a set mass difference between the two.

Lipid extracts from labeled or unlabeled cells can be resolved using the TLC technique (**Figure 4A**). It is a simple, cost effective, fast and sensitive method to qualitatively characterize lipids. Identification of broad lipid classes by TLC is simple and achieved by comparing their mobility to the standard (Urban et al., 1980). The most commonly used solvent systems to resolve sphingolipids on a TLC include: chloroform:methanol:dH_2_O (65:25:4; v/v), chloroform:methanol:4.2N NH_4_OH (9:7:2 and 40:10:1; v/v) and chloroform:methanol:acetic acid (90:2:8; v/v Mandala et al., 1995; Bodennec et al., 2000; Barreto-Bergter et al., 2004). Unlabeled sphingolipids are visualized using iodine (Palumbo and Zullo, 1987) or orcinol/resorcinol staining (Tadano and Ishizuka, 1982) and the labeled sphingolipid are visualized by autoradiography on an X-ray film (Kondo et al., 2014).

Structural composition of the purified sphingolipids can be determined using GC-EI-MS (Cacas et al., 2012). Only the volatile components that can be carried by the carrier gas (He) can be analyzed by GCMS. Most sphingolipid structures are non-volatile. For this reason, sphingolipids are hydrolyzed to release the fatty acid, sugar, and LCB components, which are then derivatized into methyl esters or TMS derivatives and analyzed by GC-EI-MS (**Figure 4A**; Christie, 1989; Matsubara and Hayashi, 1991; Johnson and Brown, 1992; Merkle and Poppe, 1994). GC-EI-MS is not a preferred technique to analyze sphingolipids because fragmentation obtained in EI-MS is not very consistent as high energy electrons are used for fragmentation, strong ionization may completely destroy the molecular ion and extensive derivatization may generate complicated spectral patterns with poor resolution (Cacas et al., 2012).

### High-performance Liquid Chromatography (HPLC)

Various sphingolipid classes can readily be separated by HPLC on both the normal phase C18 and reverse phase C8 columns. Normal phase HPLC utilizes the head group properties to obtain separation (for example, ceramides and hexosylceramides; Merrill et al., 2005). However, the reverse phase HPLC using C8 column is capable of resolving sphingolipid classes based on their hydrophobicity of carbon backbone and degree of unsaturation (for example, sphingosine and dihydrosphingosine; Rana et al., 2015). During most of the routine sphingolipid analysis the reverse phase HPLC when coupled with mass spectrometry is a powerful tool for simultaneous separation and detection of sphingolipid species. The main goal of HPLC is to chromatographically separate the sphingolipid species that cannot be resolved based on *m/z* (mass-to-charge) by the mass spectrometers, like isomeric and isobaric species, and to improve the sensitivity of detection. Several different buffer systems have been described as mobile phases (Merrill et al., 2005). The binary buffer system most frequently used as the mobile phase in sphingolipid analysis is: 2 mM ammonium formate + 0.2% formic acid in H_2_O (Buffer 1) and 1 mM ammonium formate + 0.2% formic acid in methanol (Buffer 2). A gradient elution of analytes using buffers 1 and 2 allows complete separation of sphingolipid species. Additional separation on HPLC may be achieved by lowering the flow rate of the mobile phase and changing the gradient conditions (Rana et al., 2015).

Fungal lipid extracts may contain a mixture of glucosyl- and galactosyl- ceramides (Barreto-Bergter et al., 2004). Due to a remarkable similarity in their backbone structures these lipid species are difficult to separate. These lipid species can be chromatographically separated using HILIC on silica based columns using isocratic elution (Zama et al., 2009). The elution buffer contains acetonitrile:methanol:acetic acid (97:2:1; v/v) + 5 mM ammonium acetate (Merrill et al., 2005).

### Advanced Mass Spectrometry

A wide variety of mass spectrometry platforms are now available that can be used to analyze the molecular composition of complex lipid mixtures (Köfeler et al., 2012). Mass spectrometry is a structure-based analysis of biological molecules (Wenk, 2010), and has been extensively used to characterize various sphingolipid species (Sugawara et al., 2010; Brügger, 2014; Li et al., 2014; Ogiso et al., 2014).

Several different ionization methods have been used in sphingolipid analysis, for example, the ESI (Han and Gross, 1994), MALDI (Jones et al., 2014), and FAB (Ahn et al., 2009). These ionization techniques are coupled with mass analyzers like MS/MS (Shaner et al., 2009), MS^n^ (Ito et al., 2013), TOF (Jia et al., 2015), FT-ICR (Guo et al., 2012), and QqTOF MS (Ejsing et al., 2006). The advantages and disadvantages of these techniques have been described in detail previously (Köfeler et al., 2012).

For sphingolipidomic purposes, ESI-MS/MS approach is most extensively employed. Coupled with reverse phase HPLC, ESI-MS/MS provides a simple, sensitive, and structure specific quantification of sphingolipids. Although the setup of other lipidomic platforms may be different but the basic concepts of mass spectrometry remain the same.

The primary requisition of any analytes to be analyzed by mass spectrometry depends on the fact that whether they can be readily ionized in gaseous phase without in source fragmentation (Han and Gross, 1994). ESI is a soft-ionization technique that allows the formation of intact positively or negatively charged ions that are then transmitted to the mass analyzers. In a typical ESI source, analytes in solvents are infused into the ion source via a narrow capillary needle. A high voltage, positive or negative depending upon the lipids being targeted, is applied between the tip of the needle and the inlet of mass analyzer. This results in the formation of a charged droplet which gets desolvated under high voltage, vacuum and a drying N_2_ gas, leading to the production of charged molecular ions in gaseous phase (Brügger, 2014). Sphingolipids are readily ionized in ESI source to form [M + H]^+^ molecular ions for long chain sphingoid bases, ceramides, and glycosphingolipids, or to from [M-H]^-^ molecular ions for IPC derivatives (Shaner et al., 2009).

A scheme for HPLC-ESI-MS/MS is summarized in **Figure 4B**. Different scanning approaches have developed around this triple quadrupole set up (Han and Gross, 1994; Merrill et al., 2005; Bielawski et al., 2006; Guan and Wenk, 2006; Brügger, 2014; Rana et al., 2015), and these are:

1) *Full scan* (**Figure 4C**): In a full scan, the *m/z* of molecular ions generated in ESI are recorded in the first quadrupole or the first mass analyzer. Full scan approach is widely used in non-targeted lipidomics. However, no structural information can be deduced from these scanning.
2) *Product ion scanning* (**Figure 4D**): In this mode, the first mass analyzer allows a molecular ion of specific *m/z* to pass to the collision cell. Based on the applied CE the molecular ion is fragmented into product ions using an inert collision gas (N_2_ or Ar) via RF; this process is referred as CID. The *m/z* of these fragments is recorded in the third quadrupole or the second mass analyzer. For sphingolipids, the product ion analysis provides valuable structural information about the sphingoid backbone, polar head groups and the fatty acyl attached. For example, in positive ion mode, at high CE, glucosylceramide is fragmented into a characteristic ion representing the loss of double dehydration product of sphingoid backbone (**Figure 4D**, **Table 1**). Similar ions characteristic to specific sphingolipid structures are identified and further used for lipid detection. Optimization of CE is important to obtain correct fragmentation of the molecular ions into desired daughter ions. CE required for achieving CID of different sphingolipid molecular species may vary depending upon hydroxylations, unsaturations, and carbon chain length.

**Table 1:** Sphingoid backbones and polar head groups of fungal sphingolipids.

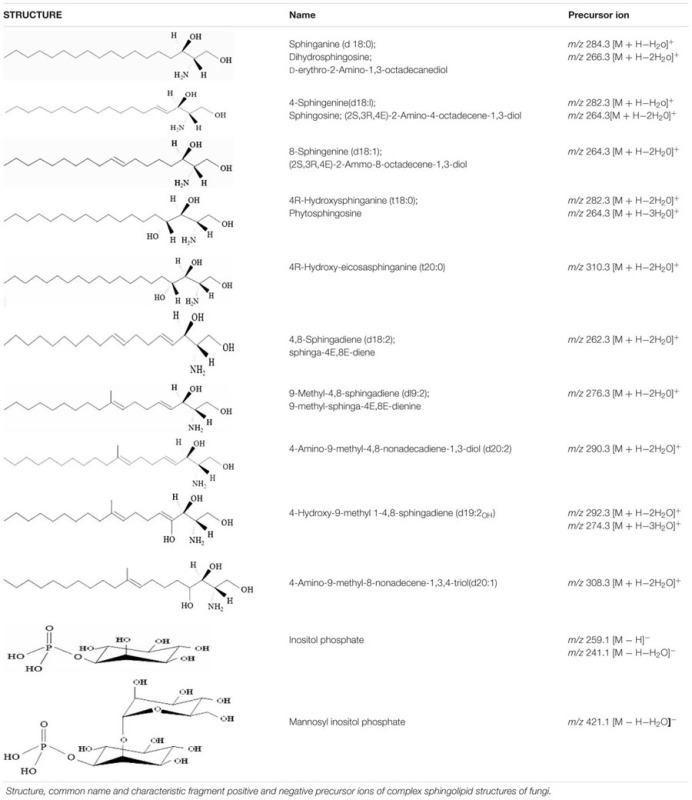

3) *Parent- or precursor-ion scanning (PREIS;* **Figure 4E**): Here, the mass analyzer records molecular ions based on their *m/z*, these ions undergo CID, and only a select daughter ion is passed into the third quadrupole. This mode is very useful in the analysis of sphingolipid species which posses a common backbone or headgroup fragment.
4) *Single-reaction monitoring (SRM;* **Figure 4F**): Only a select parent ion is recorded in the first quadrupole and undergoes CID and *m/z* of a select daughter ion is recorded in the third quadrupole. SRM’s are highly selective and very sensitive methods.
5) *Multiple-reaction monitoring (MRM;* **Figure 4G**): Several SRM reactions can be simultaneously recorded by the mass spectrometer, however, this largely depends upon the scanning speed of the instrument. MRM scanning represents a targeted lipidomics approach and is quite successfully used for the analysis of sphingolipids.
6) *Neutral loss (NL) scanning* (**Figure 4H**): Both first and third quadrupole records the *m/z*, with a constant mass offset between them. For example, a NL of 18 amu is set, which represents a loss of water molecule from the molecular ion and is characteristic of sphingolipid structures (Rana et al., 2015).

A large amount of literature containing the fragmentation pattern of different sphingolipid classes is now available. Most of these fragmentation data (product ions) were acquired using direct infusion ESI-MS/MS approaches. This, however, requires extensive oﬄine purification of sphingolipid of interest, usually using SPE (Barreto-Bergter et al., 2004). Fortunately a large amount of literature containing the fragmentation patterns of various fungal sphingolipids is available and sphingolipid class-specific fragment and precursor ions are known for most classes (Guan and Wenk, 2006). In targeted lipidomics, the unique precursor and parent ions are selected for each sphingolipid species and a MRM based method is employed for analyses (Bielawski et al., 2006).

## Analysis of Fungal Sphingolipids

By using a precursor *m/z* and product *m/z* ion pairs of molecular species of interest, an accurate detection and quantification can be done. Although the complete sphingolipidome for most fungi is yet to be determined, many sphingolipid specific backbones and polar head groups have been characterized (summarized in **Table 1**). Targeted methods using PREIS and MRM scanning methods have been developed using these fragments to trace the sphingolipid metabolism of many fungi (Bielawski et al., 2006; Guan and Wenk, 2006; Ejsing et al., 2009; Sugawara et al., 2010; Brügger, 2014; Li et al., 2014; Ogiso et al., 2014).

For example, dihydrosphingosine (d18:0) and sphingosine (d18:1) can be identified using precursor ions of *m/z* 302 and 300, and product ions of *m/z* 266 and 264, respectively, in the positive ion mode. Ions of *m/z* 266 and 264 are a result of loss of two water molecules from the sphingoid backbone. Loss of one water molecule from the backbone results ions of *m/z* 284 and 282. However, the lipid extracts may contain a mixture of d18:1 backbone with a difference in the position of double bonds, like 4-Sphingenine (major species) and 8-Sphingenine. In this situation, a prior chromatographic separation of these species is important and can be achieved by optimizing the mobile phase. The d18:0 backbone fragment ion *m/z* 266 is used to analyze dihydroceramide species while the d18:1 backbone fragment ion *m/z* 264 is used to analyze ceramide species. The phytosphingosine backbone (t18:0) is abundant in fungal lipid extract, and is constituent of phytoceramides and IPC derivatives. A precursor ion *m/z* 282 is characteristic to t18:0 backbone and results from the loss of two water molecules. Similarly, a rather less abundant t20:0 (4R-Hydroxy-eicosasphinganine) backbone is also detectable in phytoceramide and IPC structures using the positive ion precursor *m/z* 310. The presence of phosphorylinositol group/s in IPC, MIPC, and M(IP)_2_C structure often results in poor ionization efficiencies in positive ion mode. However, these lipids are readily ionized using negative ion mode and can be detected as [M–H]^-^. The precursor ion with *m/z* 241 which represents a dehydration product of the ion *m/z* 259 (the phosphorylinositol group) and can be used to analyze IPC and M(IP)_2_C species. Similarly, the precursor ion *m/z* 421 is used to analyze MIPC species (**Table 1**; Guan and Wenk, 2006; Ejsing et al., 2009; Sugawara et al., 2010).

The mass spectrometric analyses of purified lipids, especially cerebrosides, from different fungal sources have revealed several other complex backbone structures (Barreto-Bergter et al., 2011). Among these 9-methyl-4,8-sphingadiene (d19:2) is predominant in most fungal species like *Cryptococcus, Aspergillus, Candida*, and several others (Del Poeta et al., 2014). The d19:2 backbone has a characteristic fragment of *m/z* 276 that results from the loss of two water molecules. The corresponding hexosylceramide can be identified in positive ion mode using [M + H]^+^ as the parent ion. The [M + H]^+^ ions 756, 754, and 728 represent three most abundant hexosylceramides in fungi. The 2-hydroxy fatty acyl group in these structures is C_18:0_, C_18:1_, and C_16:0_. It is important to mention here that often the ions for these lipid species present themselves as Na^+^ or K^+^ adducts in positive ion or Cl^-^ adduct in the negative ion mode. Analysis of such samples requires optimization of the CE and accounting for the altered mass during the analysis. Interestingly, the differential fragmentation pattern of Cl^-^ adduct of hexosylceramide in negative ESI-MS/MS mode can be used to identify the nature of the hexose moiety (glucose or galactose). This achievable by monitoring the peak intensity ratios of two characteristic product ions: *m/z* 179 and 89. The ion intensity patterns of 179 < 89 and 179 > 89 represent galactose and glucose moiety in the hexosylceramide structure, respectively (Han and Cheng, 2005).

The 4,8-Sphingadiene (d18:2) backbone containing sphingolipid species are also common in fungi. These can be analyzed using a precursor ion *m/z* 262. Although, the *cis-* or *trans-* isomers of d19:2 and d18:2 are not reported in fungi, however, an HPLC-ESI-MS/MS based method for analysis of these was recently described using plant lipid extracts (Imai et al., 2012). Several unique sphingoid backbones in cerebroside structures have also been identified in fungi like 4-Hydroxy-9-methyl-4,8-sphingadiene (d19:2_OH_) in *Scedosporium apiospermum* and *Pseudallescheria boydii* (Pinto et al., 2002; Rollin-Pinheiro et al., 2014), 4-Amino-9-methyl-8-nonadecene-1,3,4-triol (d20:1) and 4-Amino-9-methyl-4,8-nonadecadiene-1,3-diol (d20:2) in *Cladosporium resinae* (Barreto-Bergter et al., 2011).

**FIGURE 5:**
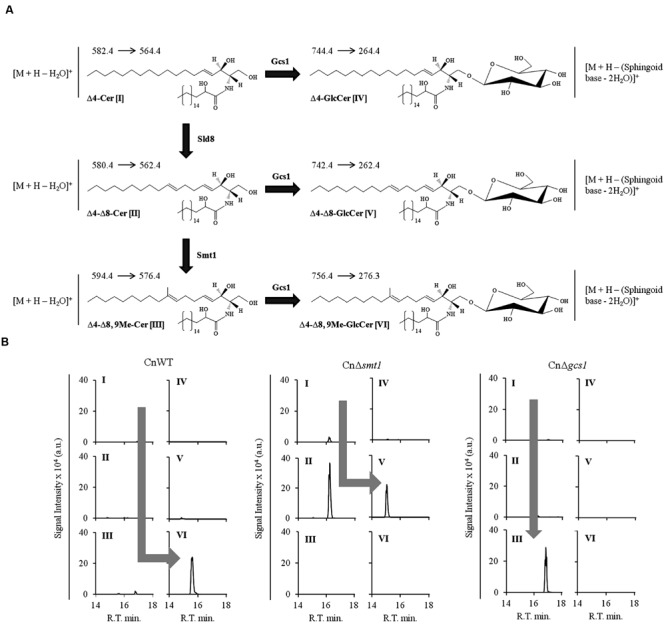
**Analysis of ceramides and glucosylceramides of *Cryptococcus neoformans* using MRM approach. (A)** Partial pathway and structures of glucosylceramide biosynthetic pathway of *C. neoformans*. Δ4-Cer [I] is converted to Δ4, Δ8-Cer [II] by Δ8-desaturase (Sld8). Δ4–8-Cer is then methylated at C9 position of sphingoid backbone to from Δ4–Δ8,9Me-Cer [III] by Smt1. A Gcs1 transfers the glucose group to sphingoid backbone of ceramide structures at C1 position forming a β1-3-glycosidic bond. This results in formation of Δ4-GlcCer (IV), Δ4, Δ8-GlcCer (V) and Δ4–Δ8,9Me-GlcCer (VI) structures. The amide-linked fatty acyl in these structures is α-hydroxylated, a key feature. The respective SRM transitions used for the identification and quantification of these structures are shown as (Parent ion→Daughter ion). **(B)** Analysis of ceramide and glucosylcamides in sphingolipid pathway mutants of *C. neoformans*. The wild type strain CnWT shows a major pool of Δ4–Δ8,9Me-GlcCer (VI), where all other structures are present in negligible amounts. The CnΔ*smt1* strain does not produce Δ4–Δ8,9Me-GlcCer (VI) structure and accumulates Δ4, Δ8-Cer [II] which is converted to Δ4, Δ8-GlcCer [IV] by Gcs1. The CnΔ*gcs1* shows an accumulation of Δ4–Δ8,9Me-Cer (III). The pathway trends are represented by gray arrows in the wild type and mutant strains.

Our ability to accurately quantify different sphingolipid structures has been efficiently used to establish the sphingolipid biosynthetic pathways in fungi. Considering that differences in the biosynthesis of various sphingolipid structures can have drastic physiological affect on fungi, it becomes necessary to understand the enzymatic steps involved therein. This has been successfully achieved for several fungal species by comparing the sphingolipid profiles of the wild type and various sphingolipid pathway mutants (Kuroda et al., 1999; Heidler and Radding, 2000; Luberto et al., 2001; Bennion et al., 2003; Ejsing et al., 2009; Cheon et al., 2012; Guimarães et al., 2014; Voynova et al., 2014). In a similar scenario, our lab worked out the glucosylceramide biosynthetic pathway of *C. neofomans* (shown in **Figure 5A**; Rittershaus et al., 2006; Singh et al., 2012). Lipid analysis of wild type *C. neofomans* strain (CnWT) showed that 1′-β-D-glucosyl-2′-*N*-hydroxyoctadecanoyl-9-methyl-4,8-sphingadiene (or Δ4–Δ8,9Me-GlcCer, detected using the SRM transition of *m/z* 756 to 276; loss of double dehydrated sphingoid base) was the most abundant cerebroside structure (**Figure 5B**, panel 1). The corresponding ceramide species 2′-*N*-hydroxyoctadecanoyl-9-methyl-4,8-sphingadiene (or Δ4–Δ8,9Me-Cer, detected using the SRM transition of *m/z* 594 to 576; water loss) was also detectable. The deletion of *SMT1* (CnΔ*smt1*) abolishes the Smt1 activity, leading to an accumulation of un-methylated ceramide and glucosylceramide structures (**Figure 5B**, panel 2). These are 2′-*N*-hydroxyoctadecanoyl-4,8-sphingadiene (or Δ4–Δ8-Cer, detected using the SRM transition of *m/z* 580 to 562; water loss) and 1′-β-D-glucosyl-2′-*N*-hydroxyoctadecanoyl-4,8-sphingadiene (or Δ4–Δ8-GlcCer, detected using the SRM transition of *m/z* 742 to 262; loss of double dehydrated sphingoid base), respectively. The Δ4–Δ8,9Me-Cer and Δ4–Δ8,9Me-GlcCer structures are absent in CnΔ*smt1* mutant. These data confirmed that methylation of ceramide by Smt1 is a pre-requisite to the formation of methylated glucosylceramide. A deletion of glucosylceramide synthase *GCS1* (CnΔ*smt1*) leads to an accumulation of Δ4–Δ8,9Me-Cer; however, all glucosylceramide structures are completely absent, suggesting that Gcs1 is the key Gcs1 in *C. neofomans*. A similar approach can also be used to study the function of Sld8, (uncharacterized in *C. neoformans*), where 2′-*N*-hydroxyoctadecanoyl-4-sphingenine (Δ4-Cer) can be detected using the SRM transition of *m/z* 582 to 564 (water loss) and 1′-β-D-glucosyl-2′-*N*-hydroxyoctadecanoyl-4-sphingenine (Δ4- GlcCer) can be detected using the SRM transition of *m/z* 744 to 264 (loss of double dehydrated sphingoid base). Thus, combined with genetic approaches, mass spectrometry becomes extremely useful in understanding the lipid metabolism.

Although, HPLC-ESI-MS/MS presents as a powerful tool for qualitative and quantitative analysis of sphingolipids in fungi as well as other biological systems. It is important to note that ESI-MS/MS only gives limited information about the structure, especially if we are looking at an uncharacterized structure. For example, it is not possible to assign positions of double bonds, hydroxyl groups, and methylation based on limited fragmentation information. Extensive MS^n^ is necessary to provide some insights into the possible arrangement of the structure (Serb et al., 2009). However, such studies require more sophisticated instrumentation with high mass resolution, high sensitivity and sub part per-million accuracy are required. Confirmation of the exact structure is only possible using NMR spectroscopy of the purified lipid species (Sarkar et al., 2015). A routine sphingolipidome profiling of fungal lipids provides limited information regarding the rate at which these structures are synthesized or the activity of the enzymes that synthesize them. More detailed information of the metabolic flux of sphingolipid species may be achievable by using the labeling approaches like stable isotope (Ecker and Liebisch) or isotopomer analysis (Hellerstein and Neese, 1999). Regardless of its limitations HPLC-ESI-MS/MS remains a method of choice for quantitative sphingolipidome profiling.

## Author Contributions

All authors listed, have made substantial, direct and intellectual contribution to the work, and approved it for publication.

## Conflict of Interest Statement

The authors declare that the research was conducted in the absence of any commercial or financial relationships that could be construed as a potential conflict of interest.

## Abbreviations

CE: collision energy
CID: collision-induced dissociation
ESI: electrospray ionization
FAB: fast atom bombardment
FT-ICR: Fourier-transform ion cyclotron resonance
GC: gas chromatography
GC-EI-MS: gas chromatography-electron impact-mass spectrometry
Gcs1: glucosylceramide synthase
HILIC: hydrophilic-interaction liquid chromatography
HPLC: high-performance liquid chromatography
IPC: inositolphosphoryl ceramide
LCB: long-chain base
MALDI: matrix-assisted laser desorption/ionization
MIPC: mannosyl-inositol phosphorylceramide
MS/MS: tandem mass spectrometry
MS^n^: multiple-stage mass spectrometry
(M(IP)_2_C: mannosyldiinositolphosphoryl ceramide
MRM: multiple-reaction monitoring
NL: neutral loss
NMR: nuclear magnetic resonance spectroscopy
PREIS: precursor-ion scanning
QqTOF MS: hybrid quadrupole time-of–flight mass spectrometer
RF: radiofrequency
SRM: single-reaction monitoring
Smt1: sphingolipid C9 methyltransferase
Sld8: Δ8-desaturase
SPE: solid-phase extraction
TLC: thin-layer chromatography
TMS: trimethylsilyl
TOF: time-of-flight
UDP: uridine 5-diphosphate

## References

[B1] Ahn Y. M. Lee W. W. Jung J. H. Lee S. G. Hong J. (2009). Structural determination of glucosylceramides isolated from marine sponge by fast atom bombardment collision-induced dissociation linked scan at constant B/E. *J. Mass Spectrom.* 44 1698–708. 10.1002/jms.1678 19824038

[B2] Barreto-Bergter E. Pinto M. R. Rodrigues M. L. (2004). Structure and biological functions of fungal cerebrosides. *An. Acad. Bras. Cienc.* 76 67–84. 10.1590/S0001-37652004000100007 15048196

[B3] Barreto-Bergter E. Sassaki G. L. de Souza L. M. (2011). Structural analysis of fungal cerebrosides. *Front. Microbiol.* 2:239 10.3389/fmicb.2011.00239 PMC3230030 22164155

[B4] Bennion B. Park C. Fuller M. Lindsey R. Momany M. Jennemann R. (2003). Glycosphingolipids of the model fungus *Aspergillus nidulans*: characterization of GIPCs with oligo-alpha-mannose-type glycans. *J. Lipid Res.* 44 2073–2088. 10.1194/jlr.M300184-JLR200 12923229

[B5] Bielawski J. Szulc Z. M. Hannun Y. A. Bielawska A. (2006). Simultaneous quantitative analysis of bioactive sphingolipids by high-performance liquid chromatography-tandem mass spectrometry. *Methods* 39 82–91. 10.1016/j.ymeth.2006.05.004 16828308

[B6] Bligh E. G. Dyer W. J. (1959). A rapid method of total lipid extraction and purification. *Can. J. Biochem. Physiol.* 37 911–917. 10.1139/o59-099 13671378

[B7] Bodennec J. Koul O. Aguado I. Brichon G. Zwingelstein G. Portoukalian J. (2000). A procedure for fractionation of sphingolipid classes by solid-phase extraction on aminopropyl cartridges. *J. Lipid Res.* 41 1524–1531. 10974060

[B8] Brügger B. (2014). Lipidomics: analysis of the lipid composition of cells and subcellular organelles by electrospray ionization mass spectrometry. *Annu. Rev. Biochem.* 83 79–98. 10.1146/annurev-biochem-060713-035324 24606142

[B9] Buede R. Rinker-Schaffer C. Pinto W. J. Lester R. L. Dickson R. C. (1991). Cloning and characterization of LCB1, a *Saccharomyces* gene required for biosynthesis of the long-chain base component of sphingolipids. *J. Bacteriol.* 173 4325–4332. 2066332 10.1128/jb.173.14.4325-4332.1991 PMC208092

[B10] Cacas J. L. Melser S. Domergue F. Joubès J. Bourdenx B. Schmitter J. M. (2012). Rapid nanoscale quantitative analysis of plant sphingolipid long-chain bases by GC-MS. *Anal. Bioanal. Chem.* 403 2745–2755. 10.1007/s00216-012-6060-1 22576656

[B11] Cheng J. Park T. S. Chio L. C. Fischl A. S. Ye X. S. (2003). Induction of apoptosis by sphingoid long-chain bases in *Aspergillus nidulans*. *Mol. Cell. Biol.* 23 163–177. 10.1128/MCB.23.1.163-177.2003 12482970 PMC140675

[B12] Cheon S. A. Bal J. Song Y. Hwang H. M. Kim A. R. Kang W. K. (2012). Distinct roles of two ceramide synthases, CaLag1p and CaLac1p, in the morphogenesis of *Candida albicans*. *Mol. Microbiol.* 83 728–745. 10.1111/j.1365-2958.2011.07961.x 22211636

[B13] Chigorno V. Sciannamblo M. Mikulak J. Prinetti A. Sonnino S. (2006). Eﬄux of sphingolipids metabolically labeled with [1-3H]sphingosine, L-[3- 3H]serine and [9,10- 3H]palmitic acid from normal cells in culture. *Glycoconj. J.* 23 159–165. 10.1007/s10719-006-7921-7 16691499

[B14] Christie W. W. (1989). *Gas Chromatography and Lipids: A Practical Guide*. Ayr: The Oily Press.

[B15] Clarke N. G. Dawson R. M. (1981). Alkaline O leads to N-transacylation. A new method for the quantitative deacylation of phospholipids. *Biochem. J.* 195 301–306. 10.1042/bj1950301 7306057 PMC1162886

[B16] Del Poeta M. Nimrichter L. Rodrigues M. L. Luberto C. (2014). Synthesis and biological properties of fungal glucosylceramide. *PLoS Pathog.* 10:e1003832 10.1371/journal.ppat.1003832 PMC3887071 24415933

[B17] Dickson R. C. Lester R. L. (2002). Sphingolipid functions in *Saccharomyces cerevisiae*. *Biochim. Biophys. Acta* 1583 13–25. 10.1016/S1388-1981(02)00210-X 12069845

[B18] Ecker J. Liebisch G. (2014). Application of stable isotopes to investigate the metabolism of fatty acids, glycerophospholipid and sphingolipid species. *Prog. Lipid Res.* 54 14–31. 10.1016/j.plipres.2014.01.002 24462586

[B19] Ejsing C. S. Moehring T. Bahr U. Duchoslav E. Karas M. Simons K. (2006). Collision-induced dissociation pathways of yeast sphingolipids and their molecular profiling in total lipid extracts: a study by quadrupole TOF and linear ion trap-orbitrap mass spectrometry. *J. Mass Spectrom.* 41 372–389. 10.1002/jms.997 16498600

[B20] Ejsing C. S. Sampaio J. L. Surendranath V. Duchoslav E. Ekroos K. Klemm R. W. (2009). Global analysis of the yeast lipidome by quantitative shotgun mass spectrometry. *Proc. Natl. Acad. Sci. U.S.A.* 106 2136–2141. 10.1073/pnas.0811700106 19174513 PMC2650121

[B21] Epstein S. Castillon G. A. Qin Y. Riezman H. (2012). An essential function of sphingolipids in yeast cell division. *Mol. Microbiol.* 84 1018–1032. 10.1111/j.1365-2958.2012.08087.x 22616608

[B22] Folch J. Lees M. Sloane Stanley G. H. (1957). A simple method for the isolation and purification of total lipides from animal tissues. *J. Biol. Chem.* 226 497–509. 13428781

[B23] Guan X. Wenk M. R. (2008). Biochemistry of inositol lipids. *Front. Biosci.* 13 3239–3251. 10.2741/2923 18508430

[B24] Guan X. L. Wenk M. R. (2006). Mass spectrometry-based profiling of phospholipids and sphingolipids in extracts from *Saccharomyces cerevisiae*. *Yeast* 23 465–477. 10.1002/yea.1362 16652392

[B25] Guimarães L. L. Toledo M. S. Ferreira F. A. Straus A. H. Takahashi H. K. (2014). Structural diversity and biological significance of glycosphingolipids in pathogenic and opportunistic fungi. *Front. Cell. Infect. Microbiol.* 4:138 10.3389/fcimb.2014.00138 PMC4174763 25309884

[B26] Guo Y. Wang X. Qiu L. Qin X. Liu H. Wang Y. (2012). Probing gender-specific lipid metabolites and diagnostic biomarkers for lung cancer using Fourier transform ion cyclotron resonance mass spectrometry. *Clin. Chim. Acta* 414 135–141. 10.1016/j.cca.2012.08.010 22906735

[B27] Haak D. Gable K. Beeler T. Dunn T. (1997). Hydroxylation of *Saccharomyces cerevisiae* ceramides requires Sur2p and Scs7p. *J. Biol. Chem.* 272 29704–29710. 10.1074/jbc.272.47.29704 9368039

[B28] Han X. Cheng H. (2005). Characterization and direct quantitation of cerebroside molecular species from lipid extracts by shotgun lipidomics. *J. Lipid Res.* 46 163–175. 10.1194/jlr.D400022-JLR200 15489545

[B29] Han X. Gross R. W. (1994). Electrospray ionization mass spectroscopic analysis of human erythrocyte plasma membrane phospholipids. *Proc. Natl. Acad. Sci. U.S.A.* 91 10635–10639. 10.1073/pnas.91.22.10635 7938005 PMC45076

[B30] Hanson B. A. Lester R. L. (1980). The extraction of inositol-containing phospholipids and phosphatidylcholine from *Saccharomyces cerevisiae* and *Neurospora crassa*. *J. Lipid Res.* 21 309–315. 6445928

[B31] Haynes C. A. Allegood J. C. Park H. Sullards M. C. (2009). Sphingolipidomics: methods for the comprehensive analysis of sphingolipids. *J. Chromatogr. B Analyt. Technol. Biomed. Life Sci.* 877 2696–2708. 10.1016/j.jchromb.2008.12.057 PMC2765038 19147416

[B32] Heidler S. A. Radding J. A. (2000). Inositol phosphoryl transferases from human pathogenic fungi. *Biochim. Biophys. Acta* 1500 147–152. 10.1016/S0925-4439(99)00097-6 10564728

[B33] Hellerstein M. K. Neese R. A. (1999). Mass isotopomer distribution analysis at eight years: theoretical, analytic, and experimental considerations. *Am. J. Physiol.* 276(6 Pt 1) E1146–E1170. 10362629 10.1152/ajpendo.1999.276.6.E1146

[B34] Heung L. J. Luberto C. Del Poeta M. (2006). Role of sphingolipids in microbial pathogenesis. *Infect. Immun.* 74 28–39. 10.1128/IAI.74.1.28-39.2006 16368954 PMC1346627

[B35] Imai H. Hattori H. Watanabe M. (2012). An improved method for analysis of glucosylceramide species having cis-8 and trans-8 isomers of sphingoid bases by LC-MS/MS. *Lipids* 47 1221–1229. 10.1007/s11745-012-3725-7 23108960

[B36] Ito E. Waki H. Miseki K. Shimada T. Sato T. A. Kakehi K. (2013). Structural characterization of neutral glycosphingolipids using high-performance liquid chromatography-electrospray ionization mass spectrometry with a repeated high-speed polarity and MSn switching system. *Glycoconj. J.* 30 881–888. 10.1007/s10719-013-9492-8 23959431

[B37] Jenkins G. M. Richards A. Wahl T. Mao C. Obeid L. Hannun Y. (1997). Involvement of yeast sphingolipids in the heat stress response of *Saccharomyces cerevisiae*. *J. Biol. Chem.* 272 32566–32572. 10.1074/jbc.272.51.32566 9405471

[B38] Jia Z. Li S. Cong P. Wang Y. Sugawara T. Xue C. (2015). High throughput analysis of cerebrosides from the sea cucumber *Pearsonothria graeffei* by liquid chromatography-quadrupole-time-of-flight mass spectrometry. *J. Oleo Sci.* 64 51–60. 10.5650/jos.ess14136 25492230

[B39] Johnson S. B. Brown R. E. (1992). Simplified derivatization for determining sphingolipid fatty acyl composition by gas chromatography-mass spectrometry. *J. Chromatogr.* 605 281–286. 10.1016/0021-9673(92)85248-R 1500464 PMC4003555

[B40] Jones E. E. Dworski S. Canals D. Casas J. Fabrias G. Schoenling D. (2014). On-tissue localization of ceramides and other sphingolipids by MALDI mass spectrometry imaging. *Anal. Chem.* 86 8303–8311. 10.1021/ac501937d 25072097 PMC4139181

[B41] Karashima T. Kajiwara K. Funato K. (2013). Metabolic labeling of yeast sphingolipids with radioactive D-erythro-[4,5-3H]dihydrosphingosine. *Bio Protoc.* 3:e862.

[B42] Kendall A. C. Pilkington S. M. Massey K. A. Sassano G. Rhodes L. E. Nicolaou A. (2015). Distribution of bioactive lipid mediators in human skin. *J. Invest. Dermatol.* 135 1510–1520. 10.1038/jid.2015.41 25668241

[B43] Kim S. Fyrst H. Saba J. (2000). Accumulation of phosphorylated sphingoid long chain bases results in cell growth inhibition in *Saccharomyces cerevisiae*. *Genetics* 156 1519–1529. 11102354 10.1093/genetics/156.4.1519 PMC1461366

[B44] Köfeler H. C. Fauland A. Rechberger G. N. Trötzmüller M. (2012). Mass spectrometry based lipidomics: an overview of technological platforms. *Metabolites* 2 19–38. 10.3390/metabo2010019 24957366 PMC3901195

[B45] Kondo N. Ohno Y. Yamagata M. Obara T. Seki N. Kitamura T. (2014). Identification of the phytosphingosine metabolic pathway leading to odd-numbered fatty acids. *Nat. Commun.* 5:5338 10.1038/ncomms6338 25345524

[B46] Kotz A. Wagener J. Engel J. Routier F. Echtenacher B. Pich A. (2010). The mitA gene of *Aspergillus fumigatus* is required for mannosylation of inositol-phosphorylceramide, but is dispensable for pathogenicity. *Fungal Genet. Biol.* 47 169–178. 10.1016/j.fgb.2009.10.001 19822220

[B47] Kuroda M. Hashida-Okado T. Yasumoto R. Gomi K. Kato I. Takesako K. (1999). An aureobasidin A resistance gene isolated from *Aspergillus* is a homolog of yeast AUR1, a gene responsible for inositol phosphorylceramide (IPC) synthase activity. *Mol. Gen. Genet.* 261 290–296. 10.1007/s004380050969 10102364

[B48] Lahiri S. Futerman A. H. (2005). LASS5 is a bona fide dihydroceramide synthase that selectively utilizes palmitoyl-CoA as acyl donor. *J. Biol. Chem.* 280 33735–33738. 10.1074/jbc.M506485200 16100120

[B49] Leipelt M. Warnecke D. Zahringer U. Ott C. Muller F. Hube B. (2001). Glucosylceramide synthases, a gene family responsible for the biosynthesis of glucosphingolipids in animals, plants, and fungi. *J. Biol. Chem.* 276 33621–33629. 10.1074/jbc.m104952200 11443131

[B50] Levery S. B. Momany M. Lindsey R. Toledo M. S. Shayman J. A. Fuller M. (2002). Disruption of the glucosylceramide biosynthetic pathway in *Aspergillus nidulans* and *Aspergillus fumigatus* by inhibitors of UDP-Glc:ceramide glucosyltransferase strongly affects spore germination, cell cycle, and hyphal growth. *FEBS Lett.* 525 59–64. 10.1016/S0014-5793(02)03067-3 12163162

[B51] Li M. Yang L. Bai Y. Liu H. (2014). Analytical methods in lipidomics and their applications. *Anal. Chem.* 86 161–75. 10.1021/ac403554h 24215393

[B52] Li S. Bao D. Yuen G. Harris S. D. Calvo A. M. (2007). basA regulates cell wall organization and asexual/sexual sporulation ratio in *Aspergillus nidulans*. *Genetics* 176 243–253. 10.1534/genetics.106.068239 17409079 PMC1893078

[B53] Li S. Du L. Yuen G. Harris S. D. (2006). Distinct ceramide synthases regulate polarized growth in the filamentous fungus *Aspergillus nidulans*. *Mol. Biol. Cell* 17 1218–1227. 10.1091/mbc.E05-06-0533 16394102 PMC1382311

[B54] Luberto C. Toffaletti D. L. Wills E. A. Tucker S. C. Casadevall A. Perfect J. R. (2001). Roles for inositol-phosphoryl ceramide synthase 1 (IPC1) in pathogenesis of *C. neoformans*. *Genes Dev.* 15 201–212. 10.1101/gad.856001 11157776 PMC312614

[B55] Luttgeharm K. D. Chen M. Mehra A. Cahoon R. E. Markham J. E. Cahoon E. B. (2015). Overexpression of *Arabidopsis* ceramide synthases differentially affects growth, sphingolipid metabolism, programmed cell death, and mycotoxin resistance. *Plant Physiol.* 169 1108–1117. 10.1104/pp.15.00987 26276842 PMC4587468

[B56] Mandala S. M. Thornton R. A. Frommer B. R. Curotto J. E. Rozdilsky W. Kurtz M. B. (1995). The discovery of australifungin, a novel inhibitor of sphinganine N-acyltransferase from *Sporormiella australis*. Producing organism, fermentation, isolation, and biological activity. *J. Antibiot. (Tokyo)* 48 349–356. 10.7164/antibiotics.48.349 7797434

[B57] Marquês J. T. Cordeiro A. M. Viana A. S. Herrmann A. Marinho H. S. de Almeida R. F. (2015). Formation and properties of membrane-ordered domains by phytoceramide: role of sphingoid base hydroxylation. *Langmuir* 31 9410–9421. 10.1021/acs.langmuir.5b02550 26262576

[B58] Matsubara T. Hayashi A. (1991). Fragmentation pathways of O-trimethylsilyl ethers of dihydroxy long-chain bases analysed by linked-scan mass spectrometry. *J. Chromatogr.* 562 119–124. 10.1016/0378-4347(91)80570-3 2026684

[B59] Merkle R. K. Poppe I. (1994). Carbohydrate composition analysis of glycoconjugates by gas-liquid chromatography/mass spectrometry. *Methods Enzymol.* 230 1–15. 10.1016/0076-6879(94)30003-8 8139491

[B60] Merrill A. H. Jr. Stokes T. H. Momin A. Park H. Portz B. J. Kelly S. (2009). Sphingolipidomics: a valuable tool for understanding the roles of sphingolipids in biology and disease. *J. Lipid Res.* 50(Suppl.) S97–S102. 10.1194/jlr.R800073-JLR200 19029065 PMC2674747

[B61] Merrill A. H. Jr. Sullards M. C. Allegood J. C. Kelly S. Wang E. (2005). Sphingolipidomics: high-throughput, structure-specific, and quantitative analysis of sphingolipids by liquid chromatography tandem mass spectrometry. *Methods* 36 207–224. 10.1016/j.ymeth.2005.01.009 15894491

[B62] Mor V. Rella A. Farnoud A. M. Singh A. Munshi M. Bryan A. (2015). Identification of a new class of antifungals targeting the synthesis of fungal sphingolipids. *MBio* 6:e00647 10.1128/mBio.00647-15 PMC4479701 26106079

[B63] Nagiec M. M. Baltisberger J. A. Wells G. B. Lester R. L. Dickson R. C. (1994). The LCB2 gene of *Saccharomyces* and the related LCB1 gene encode subunits of serine palmitoyltransferase, the initial enzyme in sphingolipid synthesis. *Proc. Natl. Acad. Sci. U.S.A.* 91 7899–7902. 10.1073/pnas.91.17.7899 8058731 PMC44511

[B64] Obeid L. M. Okamoto Y. Mao C. (2002). Yeast sphingolipids: metabolism and biology. *Biochim. Biophys. Acta* 1585 163–171. 10.1016/S1388-1981(02)00337-2 12531550

[B65] Ogiso H. Taniguchi M. Araya S. Aoki S. Wardhani L. O. Yamashita Y. (2014). Comparative analysis of biological sphingolipids with glycerophospholipids and diacylglycerol by LC-MS/MS. *Metabolites* 4 98–114. 10.3390/metabo4010098 24958389 PMC4018675

[B66] Ohnishi M. Ito S. Fujino Y. (1983). Characterization of sphingolipids in spinach leaves. *Biochim. Biophys. Acta* 752 416–422. 10.1016/0005-2760(83)90271-0

[B67] Oura T. Kajiwara S. (2010). *Candida albicans* sphingolipid C9-methyltransferase is involved in hyphal elongation. *Microbiology* 156(Pt 4) 1234–1243. 10.1099/mic.0.033985-0 20019081

[B68] Palumbo G. Zullo F. (1987). The use of iodine staining for the quantitative analysis of lipids separated by thin layer chromatography. *Lipids* 22 201–205. 10.1007/BF02537303 2437429

[B69] Pinto M. R. Rodrigues M. L. Travassos L. R. Haido R. M. Wait R. Barreto-Bergter E. (2002). Characterization of glucosylceramides in Pseudallescheria boydii and their involvement in fungal differentiation. *Glycobiology* 12 251–260. 10.1093/glycob/12.4.251 12042248

[B70] Prasad R. Ghannoum M. A. (1996). *Lipids of Pathogenic Fungi.* Boca Raton, FL: CRC Press.

[B71] Rana N. A. Singh A. Del Poeta M. Hannun Y. A. (2015). “Qualitative and quantitative measurements of sphingolipids by mass spectrometry,” in *Bioactive Sphingolipids in Cancer Biology and Therapy* eds Hannun Y. A. Luberto C. Mao C. Obeid L. M. (Cham: Springer International Publishing) 313–338.

[B72] Rittenour W. R. Chen M. Cahoon E. B. Harris S. D. (2011). Control of glucosylceramide production and morphogenesis by the Bar1 ceramide synthase in *Fusarium graminearum*. *PLoS ONE* 6:e19385 10.1371/journal.pone.0019385 PMC3084840 21559419

[B73] Rittershaus P. C. Kechichian T. B. Allegood J. Merrill A. H. J. Hennig M. Luberto C. (2006). Glucosylceramide is an essential regulator of pathogenicity of *Cryptococcus neoformans*. *J. Clin. Invest.* 116 1651–1659. 10.1172/jci27890 16741577 PMC1466548

[B74] Rodriguez-Cuenca S. Barbarroja N. Vidal-Puig A. (2015). Dihydroceramide desaturase 1, the gatekeeper of ceramide induced lipotoxicity. *Biochim. Biophys. Acta.* 1851 40–50. 10.1016/j.bbalip.2014.09.021 25283058

[B75] Rollin-Pinheiro R. Liporagi-Lopes L. C. de Meirelles J. V. Souza L. M. Barreto-Bergter E. (2014). Characterization of *Scedosporium apiospermum* glucosylceramides and their involvement in fungal development and macrophage functions. *PLoS ONE* 9:e98149 10.1371/journal.pone.0098149 PMC4039464 24878570

[B76] Sarkar A. Drouillard S. Rivet A. Perez S. (2015). Databases of conformations and NMR structures of glycan determinants. *Glycobiology* 25 1480–1490. 10.1093/glycob/cwv054 26240168

[B77] Sasaki T. (1981). Extensive radioactive labeling of glycolipids in cultured hamster fibroblasts by incubation of whole cells with UDP-[14C]glucose and UDP-[14C]galactose. *Biochim. Biophys. Acta* 666 418–425. 10.1016/0005-2760(81)90301-5 7326252

[B78] Schneiter R. Daum G. (2006). Extraction of yeast lipids. *Methods Mol. Biol.* 313 41–45. 16118423 10.1385/1-59259-958-3:041

[B79] Serb A. Schiopu C. Flangea C. Sisu E. Zamfir A. D. (2009). Top-down glycolipidomics: fragmentation analysis of ganglioside oligosaccharide core and ceramide moiety by chip-nanoelectrospray collision-induced dissociation MS2-MS6. *J. Mass Spectrom.* 44 1434–1442. 10.1002/jms.1625 19658121

[B80] Serra M. Saba J. D. (2010). Sphingosine 1-phosphate lyase, a key regulator of sphingosine 1-phosphate signaling and function. *Adv. Enzyme Regul.* 50 349–362. 10.1016/j.advenzreg.2009.10.024 19914275 PMC2862839

[B81] Shaner R. L. Allegood J. C. Park H. Wang E. Kelly S. Haynes C. A. (2009). Quantitative analysis of sphingolipids for lipidomics using triple quadrupole and quadrupole linear ion trap mass spectrometers. *J. Lipid Res.* 50 1692–1707. 10.1194/jlr.D800051-JLR200 19036716 PMC2724058

[B82] Singh A. Prasad T. Kapoor K. Mandal A. Roth M. Welti R. (2010). Phospholipidome of *Candida*: each species of *Candida* has distinctive phospholipid molecular species. *OMICS* 14 665–677. 10.1089/omi.2010.0041 20726778

[B83] Singh A. Wang H. Silva L. C. Na C. Prieto M. Futerman A. H. (2012). Methylation of glycosylated sphingolipid modulates membrane lipid topography and pathogenicity of *Cryptococcus neoformans*. *Cell. Microbiol.* 14 500–516. 10.1111/j.1462-5822.2011.01735.x 22151739 PMC3302964

[B84] Sperling P. Lee M. Girke T. Zähringer U. Stymne S. Heinz E. (2000). A bifunctional delta-fatty acyl acetylenase/desaturase from the moss *Ceratodon purpureus*. A new member of the cytochrome b5 superfamily. *Eur. J. Biochem.* 267 3801–3811. 10.1046/j.1432-1327.2000.01418.x 10848999

[B85] Sugawara T. Aida K. Duan J. Hirata T. (2010). Analysis of glucosylceramides from various sources by liquid chromatography-ion trap mass spectrometry. *J. Oleo Sci.* 59 387–394. 10.5650/jos.59.387 20513973

[B86] Tadano K. Ishizuka I. (1982). Isolation and characterization of the sulfated gangliotriaosylceramide from rat kidney. *J. Biol. Chem.* 257 1482–1490. 7056729

[B87] Ternes P. Sperling P. Albrecht S. Franke S. Cregg J. M. Warnecke D. (2006). Identification of fungal sphingolipid C9-methyltransferases by phylogenetic profiling. *J. Biol. Chem.* 281 5582–5592. 10.1074/jbc.M512864200 16339149

[B88] Ternes P. Wobbe T. Schwarz M. Albrecht S. Feussner K. Riezman I. (2011). Two pathways of sphingolipid biosynthesis are separated in the yeast *Pichia pastoris*. *J. Biol. Chem.* 86 11401–1114. 10.1074/jbc.M110.193094 21303904 PMC3064196

[B89] Urban P. F. Harth S. Freysz L. Dreyfus H. (1980). Brain and retinal ganglioside composition from different species determined by TLC and HPTLC. *Adv. Exp. Med. Biol.* 125 149–157. 10.1007/978-1-4684-7844-0-14 6767341

[B90] Voynova N. S. Mallela S. K. Vazquez H. M. Cerantola V. Sonderegger M. Knudsen J. (2014). Characterization of yeast mutants lacking alkaline ceramidases YPC1 and YDC1. *FEMS Yeast Res.* 14 776–788. 10.1111/1567-1364.12169 24866405

[B91] Warnecke D. Heinz E. (2003). Recently discovered functions of glucosylceramides in plants and fungi. *Cell. Mol. Life Sci.* 60 919–941. 12827281 10.1007/s00018-003-2243-4 PMC11138565

[B92] Wenk M. R. (2010). Lipidomics: new tools and applications. *Cell* 143 888–895. 10.1016/j.cell.2010.11.033 21145456

[B93] Zama K. Hayashi Y. Ito S. Hirabayashi Y. Inoue T. Ohno K. (2009). Simultaneous quantification of glucosylceramide and galactosylceramide by normal-phase HPLC using O-phtalaldehyde derivatives prepared with sphingolipid ceramide N-deacylase. *Glycobiology* 19 767–775. 10.1093/glycob/cwp047 19411660

[B94] Zanolari B. Friant S. Funato K. Sutterlin C. Stevenson B. J. Riezman H. (2000). Sphingoid base synthesis requirement for endocytosis in *Saccharomyces cerevisiae*. *EMBO J.* 19 2824–2833. 10.1093/emboj/19.12.2824 10856228 PMC203373

